# Production of soft unripened cheeses using acidic and salty coagulants: Investigation of technological and sensory characteristics

**DOI:** 10.1002/fsn3.3989

**Published:** 2024-04-18

**Authors:** Ahmad Soleimani, Ahmad Nasrollahzadeh, Morteza Khomeiri, Danial Dehnad, Edris Arjeh

**Affiliations:** ^1^ Department of Food Science and Technology, Faculty of Agriculture Gorgan University of Agricultural Sciences and Natural Resources Gorgan Iran; ^2^ Department of Food Science and Technology, Faculty of Agriculture Urmia University Urmia Iran

**Keywords:** acid–heat coagulation, cheese yield, organoleptic characteristic, soft cheese

## Abstract

Soft cheeses are coagulated milk products obtained through acidification or applying a combination of acids and heat. In this research, in order to improve technological characteristics, the effects of different coagulants (salt and acids) and process parameters (temperature and homogenization pressure) on the organoleptic, textural, and functional characteristics of soft (unripened) cheese were investigated. The results revealed significant differences between cheeses coagulated with acid and mineral salt regarding protein recovery, fat content, and moisture content (*p* < .05). Acidic coagulants (74%–94%) resulted in higher cheese yield compared to mineral salt (66%–88%). Texture analysis indicated that the cheese produced with acetic acid had a firmer texture, while samples treated with citric acid exhibited better cohesiveness. Cheeses produced with minerals displayed more acceptable organoleptic characteristics regarding flavor, odor, and texture. This study offers valuable technological insights into cheese production with the highest yield and maximum acceptability.

## Graphical abstract

Soft (unripened) cheeses are coagulated milk products obtained through acidification or applying a combination of acids and heat. In this research, the effects of different coagulants (salt and acids) and process parameters on the technological characteristics of soft cheese were investigated. This study offers valuable technological insights into cheese production with the highest yield and maximum acceptability.

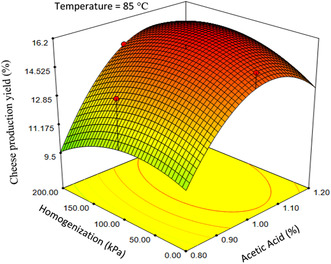

## 1 INTRODUCTION

Fresh soft (unripened) cheeses are spreadable cheeses prepared through the coagulation of milk or whey, achieved by acidification or a combination of acid and heat. These cheeses are ready for consumption immediately after their production processes. It is important to note that fresh soft cheeses differ from cheeses coagulated by rennet at a pH range of 6.4–6.6 and from fermented milk products, where most of the moisture is removed through ultrafiltration or centrifugal separation after coagulation. In these cheeses, the coagulation process occurs either near the casein isoelectric point (pH = 4.6) or at higher pH values if thermal treatment is utilized. For instance, in Ricotta cheese, coagulation occurs at pH = 6 and 75°C, involving mild acidification of heated milk through the addition of edible acids to decrease the pH to its final value (Ortiz Araque et al., 2018). Acid–heat‐coagulated cheeses account for approximately 25% of cheese production worldwide. In Iran, this type of cheese is produced without the addition of rennet, typically utilizing yogurt, and is referred to as “lactic cheese.”

Acid–heat‐coagulated (unripened) cheeses are obtained from milk following precipitation of some of its solid parts by heating of the whole milk and addition of edible acids or other suitable coagulants, leading to whey drainage (Singh et al., 2014; Wilkinson & LaPointe, 2020). A pivotal stage in cheese production is milk coagulation, resulting in curd formation through enzymatic processes, often accompanied by lactic acid production from fermentation or direct acidification of milk at elevated temperatures. These cheeses can be crafted from whole milk, skim milk, cream, whey protein, or various combinations sourced from cow or buffalo milk. Generally, cheeses produced through acid or acid–heat coagulation exhibit sour or mildly sour flavors, dependent on the type of acid used, and are typically consumed in their fresh state (Natrella et al., 2020; Phadungath, 2005). Common examples of directly acidified cheeses include Ricotta, Latin American White cheese, QuesoBlanco, Chhana, and Paneer (Farkye, 2017; Hydamaka et al., 2001). Variations among these cheeses can be attributed to differences in whey drainage methods, washing stages, cream content, and curd structure. In Cottage and Quark cheese production, the curd is segmented into predetermined sizes, whereas curd obtained through direct acidification of milk and heat treatment is usually agitated, leading to a disrupted curd structure. The standard definitions of fresh cheeses can vary from one country to another, but most are rooted in the chemical composition of the cheeses. For instance, Chhana is a traditional Indian cheese obtained from cow or buffalo milk through coagulation using sour milk, lactic acid, or citric acid, its moisture and fat should not exceed 70% and 50% of dry matter, respectively (Sahul & Das, 2010). The most critical factors impacting the flavor and yield of acid–heat‐coagulated cheese are the type of milk, the coagulant used, and the coagulation temperature. Karadbhajne and Bhoyarkar (2010) investigated the effect of ascorbic acid, citric acid, lactic acid, and tartaric acid at two levels of 2% and 4% on buffalo milk and found that using of 2% ascorbic acid resulted in the highest acceptability and textural quality. Among the various methods employed to enhance texture, flavor, and, notably, yield through the modification of traditional or introduction of novel technologies, ultrafiltration has been applied, leading to a 3.3% increase in cheese yield (Hydamaka et al., 2001).

According to surveys, these types of cheeses boast high levels of fat‐soluble vitamins, such as A and D, proteins, and minerals, while having low lactose levels, rendering them suitable for individuals with diabetes (De, 1980; Ruiz et al., 2006). Furthermore, their protein content, digestibility, and biological values (80%–86%) are noteworthy (Khan & Pal, 2011; Kumar et al., 2014).

The challenges associated with producing acid–heat‐coagulated cheeses on an industrial scale encompass the transportation of milk, cheese curd, and whey at elevated temperatures and yield losses during whey drainage from vats. Additionally, given the availability of diverse milk sources, variations in milk composition, a multitude of production techniques, and evolving consumer demands, there is a pressing need for enhanced efficiency and quality characteristics (Hydamaka et al., 2001). Most studies to date have primarily focused on refining production methods, possibly because differences in the chemical properties of acids lead to forming gels with distinct structures, necessitating comprehensive investigations into these cheeses' organoleptic and textural properties (Kumar et al., 2014). In Iran, despite the frequent consumption of these cheese types, in‐depth studies on the effects of different coagulants and milk sources on the organoleptic and textural properties of acid–heat‐coagulated chesses have been limited. Consequently, this study aims to evaluate and compare the impact of mineral salts and acids on the organoleptic/textural properties and cheese yield to provide solutions to increase cheese yield on an industrial scale while enhancing the organoleptic and textural qualities (resulting in higher absorption of milk solids) of the final product. For this purpose, the effects of acidic coagulants, including citric acid and acetic acid, will be investigated compared to mineral salt (ionic) when applied to cow's milk to achieve this objective.

## 2 MATERIALS AND METHODS

### 2.1 Materials

Different stages of this project were carried out Gorgan University of Agricultural Sciences and Natural Resources and Kalleh Dairy Co., Amol, Iran. Materials used were: milk (fat: 3.05%, non‐fat dry matter: 8.37%; pasteurization at 72°C for 15 s) from Kalleh Dairy Co. (Amol, Iran), citric acid and acetic acid bought from Shams Company (Iran), and mineral salt (SCA Co, Piacenza, Italy) and calcium chloride (Kemira, Sweden).

### 2.2 Cheese production

Milk (fat: 3.05%, nonfat dry matter: 8.37%; homogenized at 0–200 kPa) was heated to 90°C by microwave and held in water bath (WNB‐14, Memmert, Germany) for 5 min. For coagulation of milk components through coagulants, the samples were cooled to suitable coagulation temperatures (70–80°C). To produce acid–heat‐coagulated cheeses, 10% of citric acid and acetic acid solutions were prepared and added to milk at a concentration of 0.8%–1.2% (V/V), with gentle stirring. For salt‐coagulated treatments, 5% calcium chloride solution was prepared and added to milk at a concentration of 0.8%–1.2% (v/v), with gentle stirring. Milk heating was continued for 5 min to ensure complete coagulation. Before whey drainage, the curd was kept in the whey for 5 min and transferred to molds with cheesecloth. Finally, 1 kPa/cm^2^ pressure was applied for 20 min, and the prepared cheese was transferred to the cold storage (5°C) before carrying out intended chemical, mechanical/textural, and sensory evaluations on it (Singh et al., 2014).

### 2.3 Chemical experiments

#### 2.3.1 Moisture content and acidity

The cheeses were analyzed for moisture and acidity according to the methods described by AOAC (Association of Official Analytical Chemists, 2005).

#### 2.3.2 Fat content

The fat content was determined according to the Gerber–van Gulik method (Sarantinopoulos et al., 2002) and expressed as fat in dry matter.

#### 2.3.3 Protein content

The protein content of cheese samples was determined based on the macro‐Kjeldahl method (Tian et al., 2022). The conversion factor 6.38 was used to convert the obtained nitrogen into protein content.

### 2.4 Cheese yield

The yield of cheese was calculated as the amount of cheese obtained (kg) from 100 kg of milk (Carrillo‐Lopez et al., 2020).

### 2.5 Texture profile analysis

Cheese was analyzed after 48 h of storage for texture profile analysis (TPA) using a universal texture analyzer (QTS25CNS; Farnell, England) equipped with a 36‐mm‐diameter probe to measure the textural characteristics of the cheese samples. The samples, immediately after taking them out of cold storage (6°C), were cut into 20 × 20 × 20 mm^3^ and compressed using a 100 N load cell twice (at room temperature) and a crosshead speed of 75 mm/min to 50% of initial height. All samples were kept in 8% brine solution at cold storage before the analysis and the temperature was 6°C during the experiment. Cohesiveness (dimensionless), elasticity (mm), hardness (g), gumminess (g/s), chewiness (g), and compression work (the area under force‐deformation curve in the first cycle until 10 mm depth; g/s) were measured (Fox et al., 2017a).

### 2.6 Sensory evaluation

Six trained panelists, able to recognize the difference between acid–heat‐treated cheeses with different flavors and textures, of Kalleh Dairy Co. (Amol, Iran) were requested to carry out the analyses. A 5‐scale hedonic test was used for scoring texture, flavor, and odor of cheeses. The produced cheeses were cut into cubes of 20 mm diameter and were supplied to the panelists (at 20°C).

### 2.7 Statistical analysis

To determine effective factors on textural and qualitative properties of cheeses, response surface methodology and central composite designs (applied more frequently than other available designs) were used. According to the literature review, coagulation temperature, coagulant type, fat/protein ratio, and coagulation pH value are the most important factors affecting the production process of these cheeses. Hence, in this research, three variables of coagulation temperature, type and concentration of coagulant, and homogenization level were selected at three levels as mentioned in Table 1. Table 2 outlines the RSM design with 17 treatments used in this research to apply three levels of independent variables with three replications at the central point and three replications for each chemical/textural test of cheese. Quantity and type of experiments were the same for each one of coagulants, as indicated in Table 2, but for salt‐coagulated treatments, levels of salt were the half of mentioned concentrations. Selecting each level of independent variables was based on the literature review and previous experiences. Mean comparison of results was carried out at the confidence level of 95% (*p* < .05). Design expert (Version.7.0.0) software was used for fitting of data and depicting of figures.

**TABLE 1:** Variables levels of lactic cheese production for each coagulant type.

Factor type	Level 1	Level 2	Level 3
Temperature (°C)	80	85	90
Coagulants amount (%)	0.8	1	1.2
Homogenization pressure (kPa)	0	100	200

**TABLE 2:** Coded RSM design (17 treatments) used for each coagulant type in this research.

Treatment number	Temperature	Coagulant level	Homogenization pressure
1	0	0	0
2	0	0	0
3	1	−1	1
4	−1	−1	1
5	1	1	1
6	−1	0	0
7	1	1	1
8	1	1	−1
9	1	−1	−1
10	0	−1	0
11	0	0	−1
12	0	1	0
13	1	0	0
14	−1	−1	−1
15	0	0	1
16	−1	1	1
17	0	0	0

## 3 RESULTS AND DISCUSSION

In this study, a central composite design was used to optimize the effects of various factors on the production process of acid–heat‐coagulated cheeses. The effects of different processing variables on cheese yield and quality were determined, and the average values are inserted in Table 3.

**TABLE 3:** Results of physicochemical properties for different treatments (acetic acid [AA], citric acid [CA], mineral salt [MS]) of lactic cheese.

Treatment	Moisture (%)			Fat (%)			Protein (%)			Production yield (%)			Protein recovery (%)		
	AA	CA	MS	AA	CA	MS	AA	CA	MS	AA	CA	MS	AA	CA	MS
1	54.0	54.6	58.9	48.7	50.6	46.6	37.6	34.6	36.1	15.3	15.6	15.7	91	92	86
2	50.7	51.6	59.2	47.7	53.5	45.9	36.1	33.3	36.0	15.2	15.7	15.5	90	91	86
3	52.8	55.9	56.4	48.7	51.1	46.7	37.9	37.0	38.8	14.2	14.0	15.4	83	74	78
4	0.00	0.00	63.8	0.00	0.00	63.8	0.00	0.00	40.7	0.00	0.00	13.5	0.00	0.0	68
5	48.0	50.7	61.4	48.3	45.7	44.3	37.2	38.8	37.6	12.2	14.0	15.8	90	91	77
6	53.9	50.3	63.7	45.5	47.7	39.7	34.7	36.8	34.1	14.4	13.7	12.9	89	92	83
7	51.3	51.7	58.6	48.6	48.2	46.2	36.6	38.5	37.2	13.7	13.8	15.8	92	93	88
8	47.9	51.5	54.5	47.0	45.3	47.1	37.4	36.7	37.7	11.7	13.6	15.7	93	94	85
9	54.9	58.6	54.7	47.7	45.8	48.3	37.8	37.2	37.3	16.1	16.2	15.4	85	86	87
10	54.7	55.7	59.9	47.7	48.6	46.9	36.3	36.4	37.0	14.2	15.9	16.3	90	91	85
11	56.2	54.4	57.9	48.0	48.3	46.6	37.2	37.0	35.8	15.6	15.4	15.3	91	91	84
12	50.2	55.0	56.9	48.0	47.4	48.1	35.4	35.9	37.0	13.1	11.8	14.3	93	94	86
13	50.2	51.1	57.1	44.8	48.8	46.6	35.8	36.8	37.6	14.5	13.6	16.4	92	93	87
14	0.00	0.00	62.6	0.00	0.00	41.3	0.00	0.00	34.4	0.00	0.00	14.6	0.00	0.0	66
15	52.5	54.9	59.5	48.4	48.7	45.2	36.2	35.3	37.1	14.7	15.0	15.6	90	92	75
16	52.4	54.0	61.8	48.7	48.3	45.4	36.0	36.5	36.2	13.8	15.1	17.9	89	91	82
17	51.2	51.9	58.1	47.4	50.5	46.5	35.5	33.4	36.4	15.2	15.1	15.9	91	91	87

### 3.1 Moisture content

The response surface analysis revealed a significant inverse relationship between cheese moisture content temperature and acid concentration. Furthermore, the coagulation temperature of milk exhibited a noteworthy impact on cheese moisture content (*p* < .05). The analysis of variance illuminated the comprehensive influence of processing variables on the regression model of moisture content. Specifically, concerning acidic coagulants, the linear effects of temperature and acid levels exhibited significance, whereas, for mineral salts, the linear effects of temperature and homogenization level were found to be significant.

**FIGURE 1:**
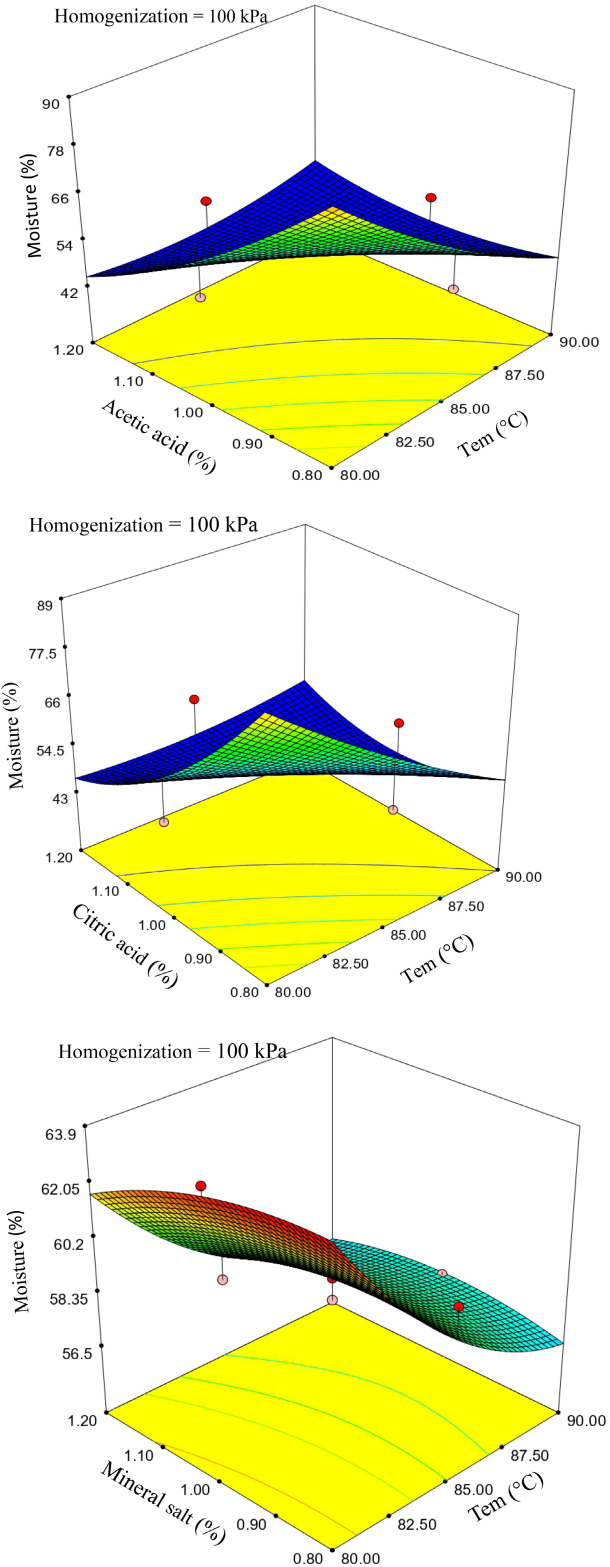
Response surface plats for the effect of temperature and coagulant (acid/salt) on the moisture content of coagulated cheese.

The pH values of cheeses treated with acid ranged between 5.7 and 6.0 and were not contingent upon the type of acid utilized. The effects of temperature and coagulant level on moisture content are prominently depicted in Figure 1: an increase in temperature from 80 to 90°C resulted in a significant reduction in cheese moisture content (*p* < .05). This decrease in the moisture content of cheese curd is likely attributed to the diminished water‐holding capacity of whey proteins due to the elevated temperature.

Teo et al. (1996) ascribed the reduction in moisture content of acidic casein curd to enhanced interactions among protein molecules due to temperature escalation, along with the loss of hydration properties due to whey protein denaturation. As illustrated in Table 3, cheeses treated with ionic salts exhibited higher moisture content than those coagulated with acids. Furthermore, cheeses produced through the influence of citric acid demonstrated higher moisture content than samples treated with acetic acid. The elevated moisture content in citric acid‐treated samples can be attributed to its milder impact on milk proteins. Citric acid possesses a higher pKa and lower hydrogen ion concentration per unit weight, resulting in a slower release of hydrogen ions from solutions. This characteristic leads to milder denaturation of milk proteins and more excellent hydration capabilities (Khan et al., 2014).

Conversely, salt ions are pivotal in establishing cross‐links within the curd structure. They facilitate compression and coagulation of milk proteins by neutralizing protein charges, thereby enhancing the moisture content of cheese (Guo et al., 2003). Additionally, the results indicated that increasing acid concentration resulted in a reduction of moisture content in cheese. Khan and Pal (2011) and Kinjal et al. (2015) have previously reported an inverse relationship between coagulant concentration and the final moisture content in cheese. Consequently, coagulant concentration can serve as an indicator of moisture content decrease or increase in cheese.

### 3.2 Fat and protein recovery

In the case of salt‐coagulated cheeses, only the linear effect of temperature on fat recovery exhibited significance (*p* < .0003), whereas for acid‐coagulated counterparts, both the linear effects of temperature and acid concentration were statistically significant (*p* < .05).

**FIGURE 2:**
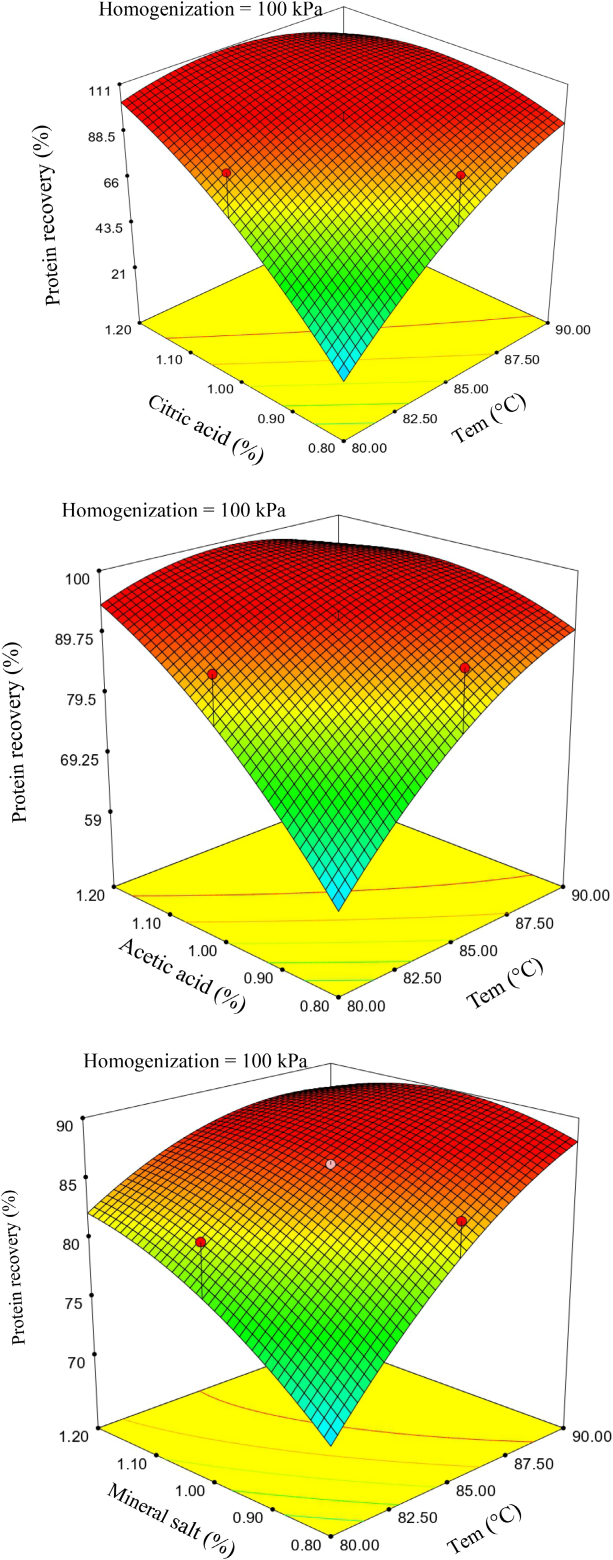
Response surface plots for the effect of temperature and coagulant (acid/salt) on the protein recovery of coagulated cheese.

The linear effects of temperature and coagulant concentration displayed significance concerning protein recovery for all three coagulants. As evident in Figure 2, coagulant concentration exerted a more pronounced influence on protein recovery than the other two variables. This observation implies that the microscopic structure of these cheese varieties was significantly impacted by the type of coagulant employed. The results concerning the recovery of milk solid contents indicated that citric acid coagulant yielded the highest fat and protein recoveries. Hydamaka et al. (2001) previously reported that the choice of acid was pivotal in influencing cheeses' compositional, organoleptic, and yield aspects. Notably, the interaction effect of coagulant concentration and homogenization degree on fat recovery did not exhibit statistical significance.

A comprehensive examination through response surface analysis revealed that increasing the homogenization pressure to 100 bar and a temperature elevation facilitated fat recovery. However, a higher increase in homogenization degree led to only a minimal decrease in fat recovery, particularly at elevated temperatures, where this effect was less pronounced. In contrast, protein loss in whey was more substantial for samples treated with salt than those treated with acid. Elevated temperature and coagulant concentration both contributed to enhanced fat and protein recovery. This indicates that the mechanism of action of acid coagulants during milk protein coagulation differs from that of salt coagulants.

As acid coagulants reduce the pH value of milk, they induce spatial destabilization of casein micelles by reducing electric repulsion and Van der Waals interactions. Consequently, they facilitate the gradual removal of calcium phosphate from casein micelles as soluble calcium salt, ultimately resulting in protein compression. Thus, a substantial and coherent structure is formed by the compression of casein micelles in conjunction with a network comprising milk fat, soluble solids, other colloids, and whey proteins (Fox et al., 2017b).

As depicted in Figure 2, increasing temperature and coagulant concentration led to a reduction in protein recovery. A similar study conducted by Khan et al. (2014) on cheeses manufactured using different coagulants established that the augmentation of these two variables induced protein denaturation, significantly impacting protein binding and resulting in protein loss as particles in whey. Consequently, it is advisable to utilize a moderate level of coagulant concentration to maximize the recovery of milk solid content.

### 3.3 Cheese yield

**FIGURE 3:**
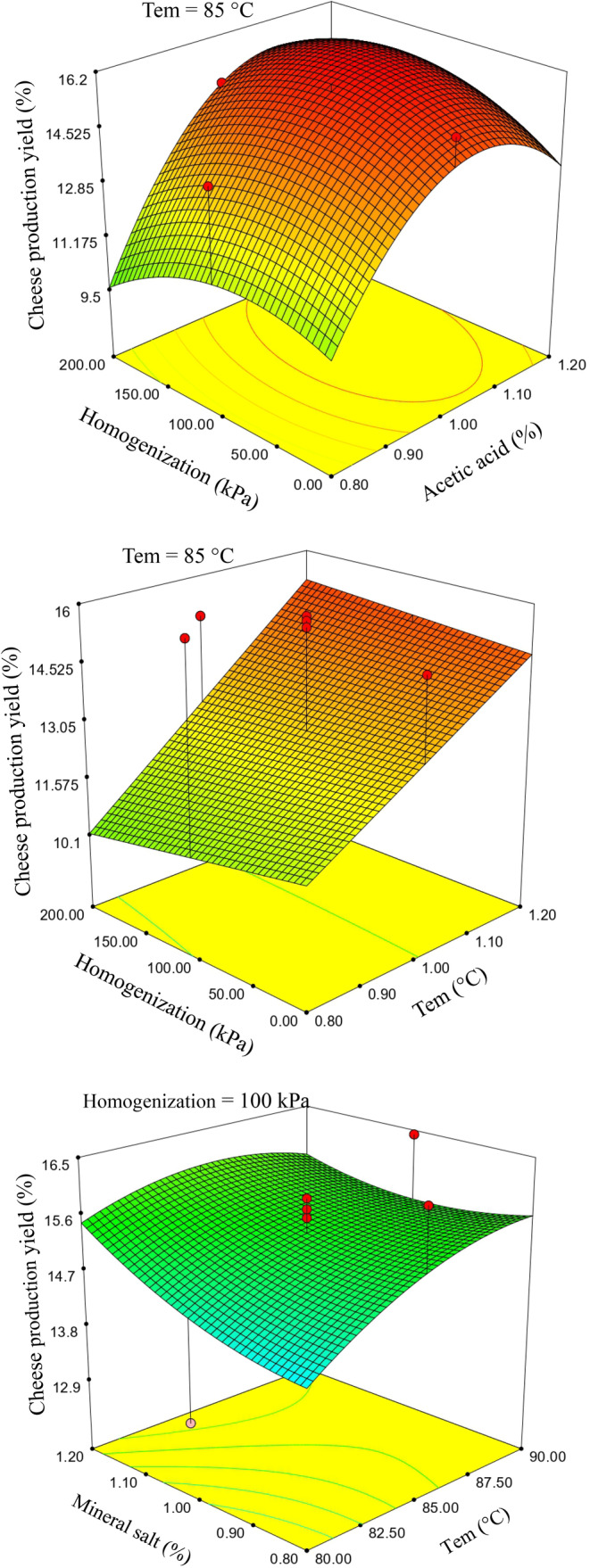
Response surface plots for the effect of temperature and coagulant (acid/salt) on the cheese production yield.

In the context of acid coagulants, the sole significant influencing factor on cheese yield is the linear impact of temperature and coagulant concentration (Figure 3). The mineral salt coagulant exhibits the highest postproduction yield due to its elevated moisture content. As evidenced in Table 3, the protein content in the dry matter of cheese produced by acid coagulants surpasses that of salt coagulants. This augmented protein and milk solid constituent recovery can be attributed to higher temperatures, increased coagulant concentration, and reduced moisture content. The diminished protein content in the dry matter of acid‐coagulated cheeses can be attributed to the lower recuperation and higher moisture content of milk solid constituents, leading to decreased protein levels per unit of cheese weight (Khan et al., 2014). The highest mass recovery for cheeses (postimmersion in brine for 24 h and subsequent water absorption) is associated with acid coagulants when compared to salt coagulants, given their nearly equivalent moisture content. The regulation of acid levels employed in acid‐coagulated cheeses results in a range of pH values from 5.60 to 5.99. The adjustment of milk pH significantly impacts the composition, recovery, and cheese yield. Therefore, the lower cheese yield in samples treated with acetic acid, compared to those treated with citric acid, can be attributed to increased syneresis and whey loss from the curd, stemming from more significant protein compression (Chandan, 1991).

Among the acid‐coagulated cheeses, treatments numbered 1, 2, 9, 10, and 11, and among the salt‐coagulated cheeses, treatments numbered 1, 5, 10, and 16 resulted in the highest mass recovery. However, their moisture contents exceeded those of the other treated samples (Table 3). Homogenization up to 100 bar, when compared to the absence of homogenization, increased cheese yield. Typically, homogenized milks are not employed in producing enzymatic cheeses due to their adverse effects on curd structure properties. Surveys have indicated that the use of homogenized milk in the production of acid–heat cheese reduces fat loss in whey but has a negative impact on curd hydration. Figure 3 illustrates that the optimal conditions for producing this type of cheese using acid coagulants (concerning cheese yield) are achieved by selecting the second level for coagulant concentration and homogenization pressure while keeping the temperature higher than the second level nonetheless since sample weight is not the sole critical factor in assessing and determining optimal conditions, textural and flavor indices are also employed for this purpose.

### 3.4 Texture

Response surface analysis showed that the linear effects of temperature and coagulant level on the whole textural properties of cheeses were significant for all three coagulants types (*p* < .05). Linear effect of homogenization degree was significant on cohesiveness (*p* < .01) of cheese for acid‐coagulated samples and on gumminess (*p* < .006), chewiness (*p* < .002), and cohesiveness (*p* < .04) for mineral salt‐coagulated ones.

The work required to deform the samples increased with escalating temperature levels and coagulant concentrations. Compression work, which measures the energy needed to deform samples under the applied compressional force, indicated that treatment number 8 demanded the highest energy for deformation, reducing the dimensions by 50% of their initial size in the case of all three coagulants. This distinction was statistically significant compared to other treatments. Notably, acetic acid exhibited a more pronounced effect in increasing compression work than other coagulants.

**FIGURE 4:**
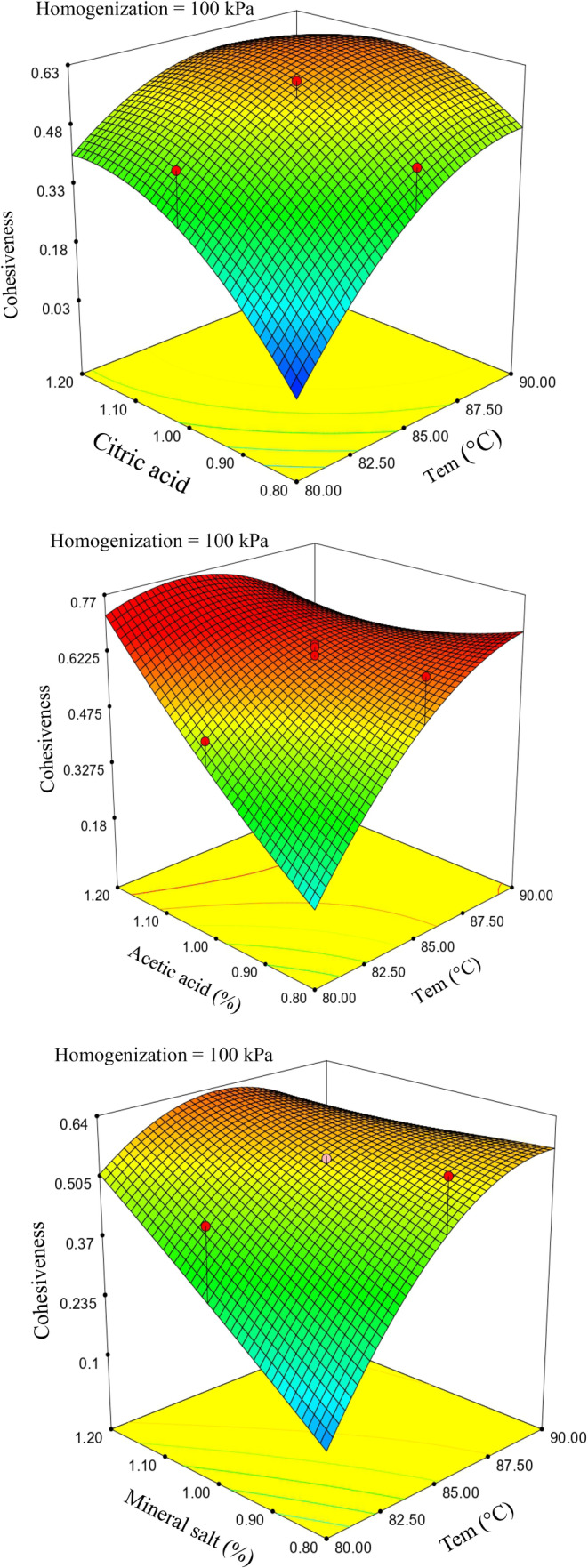
Response surface plots for the effect of temperature and coagulant (citric acid, acetic acid, or mineral salt) on th texture of coagulated cheese.

Cohesiveness reflects the rate of deformation experienced by a sample during compression by molar teeth before tearing, and it is contingent on the strength of the internal bonds constituting the product's body. As depicted in Figure 4, the highest cohesiveness in cheese samples was achieved with citric acid coagulants. In this context, the interactive effects of temperature and coagulant levels on texture proved significant. Variations most significantly influenced cohesiveness in coagulant levels. According to McCarthy et al. (2017), the level and type of coagulant are effective on texture and body of the product through changing structure of proteins, and as a result, their bonding, water holding capacity, gel formation capacity.

Furthermore, the response surface analysis revealed that treatments characterized by the third level of temperature, the second level of coagulant amount, and the homogenization level for mineral salt‐coagulated cheeses, as well as the second level of each variable factor for acid‐coagulated samples, yielded the most favorable textural and organoleptic characteristics.

### 3.5 Organoleptic properties

Response surface analysis revealed that neither of the variables significantly impacted the organoleptic properties of cheeses produced using acetic acid coagulants. However, for citric acid‐treated samples, both flavor and odor properties were influenced by temperature and coagulant type, and their linear and interaction effects were significant. In the case of salt‐treated samples, the linear effects of temperature and homogenization level were significant factors affecting the flavor of cheeses, and the linear effect of temperature was also significant for odor. The results of the organoleptic properties of samples treated with all three types of coagulants are presented in Table 4. The scores provided by the panelists revealed that cheeses produced with citric acid treatment had the lowest acceptability in flavor, odor, and texture compared to the other coagulants. This outcome is likely attributed to differences in the biochemical composition of cheeses produced using citric acid. Increasing the coagulant level and temperature, and thus their interaction, led to decreased flavor and odor scores in acid‐coagulated cheeses. Research indicates that lower acid and temperatures result in a slower and milder coagulation process due to more comprehensive coagulant activity throughout the milk volume, ultimately improving odor and flavor characteristics (Khan et al., 2014).

**TABLE 4:** Effects of different treatments on organoleptic properties of lactic cheese prepared by citric/acetic acid or heat coagulation.

Treatment	Acetic acid				Citric acid				Mineral salt			
	Flavor	Odor	Texture	Total	Flavor	Odor	Texture	Total	Flavor	Odor	Texture	Total
1	2.9	3.1	3.6	3.2	3.0	2.2	3.1	2.8	3.5	3.2	3.8	3.5
2	3.0	3.2	3.8	3.3	3.2	2.5	2.8	2.7	3.1	3.2	3.8	3.4
3	2.0	2.4	3.2	2.5	2.6	2.4	2.2	2.4	3.5	4.0	4.0	3.8
4	0.0	0.0	0.0	0.0	0.0	0.0	0.0	0.0	2.5	1.4	1.2	1.7
5	3.2	3.2	3.7	3.4	2.6	2.2	2.3	2.4	3.0	4.0	2.7	3.2
6	3.0	2.6	3.0	2.9	2.2	2.4	2.4	2.3	2.2	2.1	1.9	2.1
7	2.0	2.2	2.9	2.4	1.8	1.6	2.1	1.8	2.0	3.1	3.1	2.7
8	2.8	3.2	2.6	2.9	2.8	2.4	2.8	2.7	4.0	4.6	3.7	4.1
9	4.0	4.0	3.9	4.0	2.2	2.4	2.7	2.4	4.6	4.1	3.0	3.9
10	3.4	3.4	3.8	3.5	2.2	1.8	1.4	1.8	3.0	3.0	3.1	3.0
11	3.2	2.8	3.7	3.2	2.2	2.6	2.7	2.5	3.1	3.0	4.0	3.4
12	2.6	2.4	2.7	2.6	2.4	2.8	2.8	2.7	2.1	2.0	1.9	2.0
13	2.0	2.2	1.6	1.9	2.6	2.2	2.7	2.5	3.0	3.1	4.8	3.6
14	0.0	0.0	0.0	0.0	0.0	0.0	0.0	0.0	1.9	1.3	1.1	1.4
15	2.8	3.2	3.2	3.1	2.4	2.4	3.0	2.6	3.1	3.1	4.0	3.4
16	2.6	3.2	2.4	2.4	2.8	2.6	3.1	2.8	3.0	2.3	2.0	2.4
17	3.0	3.2	3.5	3.2	3.0	2.6	2.7	2.8	3.2	3.2	4.5	3.6

Conversely, for mineral salt‐treated cheeses, raising the temperature and coagulant level worsened the flavor and intensified the odor, with temperature having a more pronounced effect. This observation underscores the paramount importance of temperature in producing cheeses using mineral salt coagulants. Lower scores in terms of textural acceptability for cheeses produced using all three types of coagulants at higher temperatures and coagulant levels may be attributed to the increased denaturation of milk proteins, affecting their secondary interactions with each other and water molecules. This results in harder cheeses with higher dry matter content and lower scores (Kumar et al., 2014).

As depicted in Table 3, among cheeses produced via acid‐coagulated processes, treatment number 9 yielded the highest cheese yield at 16%, while treatment number 16 yielded the highest cheese yield at 17.85% among mineral salt‐treated samples. Consequently, in terms of cheese yield, mineral salt led to higher percentages; however, these cheeses had significantly higher moisture content and lower protein or solid content recovery.

The optimum production conditions for the flavor aspect in cheeses produced using acid coagulants were achieved at the third level for all three variables. In contrast, the optimum conditions for cheeses produced with salt treatment were at the third level for temperature and the second for coagulant level and homogenization pressure. Therefore, the optimal production conditions for organoleptic aspects differed considerably from those for weight or recovery indices. If flavor and odor are considered the primary indices, comparing average results for organoleptic properties indicates that the optimal production conditions were achieved with treatment number 2 for acid‐coagulated samples and treatment number 13 for salt‐coagulated samples. Panelists expressed that these samples had desirable textures, while some samples were described as soft or with unsatisfactory hardness. Hence, treatment number 2 is recommended with half the homogenization pressure for the production of acid‐coagulated cheeses, and treatment number 13 is recommended without homogenization for the production of salt‐coagulated samples.

According to the response surface analysis results, an increase in the homogenization level of samples resulted in a softer texture and less pronounced flavor in acid–heat cheeses.

## 4 CONCLUSIONS

Acid–heat cheese is a fresh type of cheese produced through milk coagulation. This coagulation occurs via direct chemical acidification, culture acidification, or chemical and high‐temperature treatment. The production of these cheeses varies across different countries due to the utilization of diverse techniques and coagulant types. Consequently, there is a pressing need for standardized methods in their production.

This research has revealed that cheeses produced using mineral salts exhibit increased hardness and cohesiveness compared to those coagulated with acids. However, it should be noted that these mineral salt‐coagulated cheeses experience higher protein and fat losses in their whey compared to their acid‐coagulated counterparts. As a result, the quality of acid–heat cheeses is contingent upon the type of coagulant employed and the specific processing procedures, particularly the thermal treatment of milk.

Nonetheless, it is essential to consider that producers primarily select acids for acid–heat cheese production based on their potential to enhance the qualitative attributes of the final product. For instance, applying 1% acetic acid has proven effective in producing acid–heat‐coagulated cheeses with a high yield, appropriate recovery of milk solid contents, and desirable organoleptic properties. The outcomes of this study can serve as a valuable reference for establishing national standards for these commonly used cheeses in Iran.

## AUTHOR CONTRIBUTIONS

**Ahmad Soleimani:** Data curation (equal); formal analysis (equal); software (equal); writing – original draft (equal). **Ahmed Nasrollahzadeh:** Data curation (equal); formal analysis (equal); methodology (equal); writing – original draft (equal). **Morteza Khomeiri:** Supervision (equal); validation (equal); visualization (equal). **Danial Dehnad:** Methodology (equal); validation (equal); visualization (equal). **Edris Arjeh:** Software (equal); visualization (equal); writing – review and editing (equal).

## FUNDING INFORMATION

None.

## CONFLICT OF INTEREST STATEMENT

The authors have declared no conflict of interest.

## ETHICS STATEMENT

This article does not contain any studies with human or animal subjects.

## Data Availability

Data will be made available on request.
